# Ground Strength Test Technique of Variable-Camber Wing Leading Edge

**DOI:** 10.3390/biomimetics9080467

**Published:** 2024-08-01

**Authors:** Shanshan Li, Xianmin Chen, Zhigang Wang, Yuanbo Liang

**Affiliations:** National Key Laboratory of Strength and Structural Integrity, Aircraft Strength Research Institute of China, Xi’an 710065, China; chenxm007@avic.com (X.C.); wangzg022@avic.com (Z.W.); liangyuanbo_22@163.com (Y.L.)

**Keywords:** variable-camber wing, leading edge, motor function, strength test, cooperative loading, morphological reconstruction

## Abstract

Morphing wing technology is crucial for enhancing the flight performance of aircraft. To address the monitoring challenges of full-scale variable-camber leading edges under flight conditions, this study introduces a ground-based strength testing technique aimed at precisely evaluating the deformation patterns and structural strength during actual operation. Firstly, the motion characteristics of the variable-camber leading edge were analyzed using numerical simulation based on kinematic theory. Secondly, a tracking loading test rig was designed and constructed to simulate the actuated deformation and aerodynamic loads of the leading edge. Next, mechanical boundary numerical simulation was then utilized to predict the motion trajectories of loading points on the upper and lower wing surfaces, and a multi-point coordinated control system was developed to achieve accurate experimental control. Finally, a multi-sensor iterative method was employed to ensure loading precision throughout the testing process. A case study was conducted using a leading edge test piece from a specific commercial aircraft. The results indicated that in the motion test of the variable-camber leading edge, the average error of the deflection angle was 4.59%; in the strength test, the average errors in the magnitude and direction of the applied load were 0.54% and 0.24%, respectively. These findings validate the effectiveness of the proposed technique in simulating the flight conditions of deforming wings and accurately obtaining the leading edge shape change curve, deformation accuracy curve, and strain curves of the upper and lower wing surfaces under deflection angles. Furthermore, this paper compares the deformation accuracy of different testing methods under test conditions, providing scientific evidence and technical support for the testing and evaluation of variable-camber leading edges.

## 1. Introduction

Variable-camber leading edges represent an advanced aerospace design that emulates the continuous and smooth form of a bird’s wings. By dynamically adjusting the wing surface shape, these designs optimize aerodynamic performance, reduce drag, and enhance lift, thereby improving the fuel efficiency and range of aircraft. This design not only embodies the sophisticated concept of biomimicry but also stands at the forefront of technological advancements in the field of aeronautical engineering [1,2,3]. Among the various approaches to enhancing aerodynamic efficiency, morphing wing technology is of paramount importance. Its goal is to maintain the structural integrity of the aircraft while actively and precisely deforming the wings to adapt to the requirements of real-time flight missions and flow field conditions, thereby sustaining the optimal shape [4]. To date, extensive research has been conducted by scholars on the materials for variable-camber wings, including corrugated composites [5], shape-memory alloys [6,7,8], polymers, and composites [9,10]. The aerodynamic performance of variable-camber wings significantly influences the endurance and safety of aircraft. Consequently, numerous scholars have conducted mechanical analyses of variable-camber wings [11,12,13,14,15,16], thereby elucidating the stress states experienced by these wings. Research has revealed that enhancing their aerodynamic performance is an effective technique for reducing fuel consumption [17]. To better meet the demands of flight missions, scholars have delved deeply into aerodynamic performance [18,19,20], primarily focusing on increasing lift at low speeds and the lift-to-drag ratio to improve aerodynamics. Concurrently, extensive research has been conducted on lightweight actuators [21,22,23,24,25], with a focus on the application of various algorithms such as PI control, fuzzy PID, and artificial neural networks in the design of compensators. In addition, many scholars have explored the design of variable-camber wing trailing edge structures based on the concept of flexible structures, employing load path methods [26,27,28,29,30] and NSGA-II [31] for the topological optimization of flexible structures. As research progresses, the cutting edge of variable-camber wing studies is trending towards addressing practical engineering challenges, namely aerodynamic elasticity, fatigue durability, bird-strike resistance, and de-icing issues [16].

To rapidly integrate variable-camber wings into models, extensive experimental validations have been conducted both domestically and internationally. The German Aerospace Center has conducted extensive research on variable wings, covering structural design and optimization [32] and functional verification of target deflection angles. Non-contact measurement and strain gauge measurement were employed to measure the strain of flexible skins [33]. The deformation of flexible skins was reconstructed, with plans for ground bending tests [34]. The Italian Aerospace Research Center (CIRA) conducted experimental studies on the reconstruction of deforming wing trailing edges in wind tunnels [35]. Between 2010 and 2015, the European SARISTU project was implemented. This project involved the design and manufacture of a full-scale wing with a span of 4.7 m, including the leading edge, trailing edge, and variable-camber winglets. The Central Aerohydrodynamic Institute (TsAGI) in Russia conducted experiments on the deformation functions of variable-camber wing leading and trailing edges in the T-104 wind tunnel [16]. The Chinese Aircraft Strength Research Institute has been closely tracking the design, optimization, and experimental validation of variable-camber wings, conducting meticulous work on the design optimization of variable-camber wing leading edges [36,37,38,39,40]. However, the aforementioned experiments primarily assessed the wing components under fixed conditions and did not address the flight conditions experienced by full-scale deforming wings during operation. Yet, aircraft operation encounters various complex conditions, which can lead to a decline in aerodynamic performance, compromised flight stability, and reduced structural strength under different conditions. Additionally, there is a strong interdependence among the components, and the impact of complex conditions on each deforming wing system is not well understood, significantly increasing flight safety risks. Therefore, conducting research on the deformation and stress of variable-camber wings under different conditions and assessing the deformation functions and strength of the wings are of great significance for enhancing the overall safety of aircraft.

**Figure 1:**
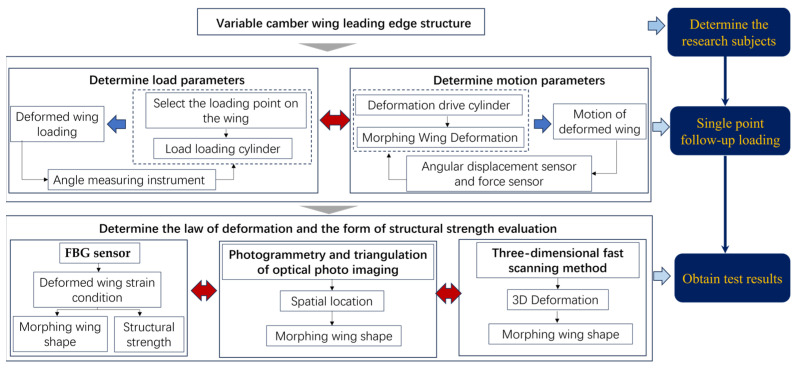
Experimental plan flow chart.

In this paper, the deformation function and load capacity verification of the leading edge of a full-scale variable-camber wing were studied. By establishing an accurate mathematical model, a followed-up load test apparatus was designed. A multi-point cooperative control system was developed through motion simulation and sensor network design technology. A distributed sensor monitoring network was formed by many advanced measurement methods and structural configuration reconstruction technology. The motion function and structural strength of the leading edge structure of the variable-camber wing were verified and evaluated by tests. The ground strength testing plan for the variable-camber wing leading edge is depicted in Figure 1.

## 2. Variable-Camber Wing Shape Theory

### 2.1. Theoretical Model of Variable-Camber Wings

The design of the variable-camber leading edge emulates the continuous and smooth variation found in the wings of birds in nature, thereby providing the aircraft with superior aerodynamic performance and flight adaptability. Unlike traditional wings, the deflection of these variable-camber wings is achieved through a series of precise mechanical movements. This process is typically activated by deformation-driven actuation cylinders that engage a linkage mechanism, thereby transferring motion to the wing leading edge. This results in deformation of the composite skin and reinforcement components to achieve the desired deflection angle. Therefore, the precise deflection of the variable-camber wing is closely related to its shape contour. To obtain the target shape contour of the variable-camber wing, this paper employs a target function based on the weighted least squares error (*WLSE*), as shown in Equation (1).

(1) $$WLSE=\sum_{i=1}^{n}w_{i}\frac{d_{i}}{n}=\sum_{i=1}^{n}w_{i}\frac{\sqrt{{\left(x_{i}-{x_{i}}^{*}\right)}^{2}+{\left(y_{i}-{y_{i}}^{*}\right)}^{2}}}{n}$$

In the equation, *n* represents the number of monitoring points; *w_i_*, *d_i_*, (*x_i_*,*y_i_*), and (x_i_*,y_i_*) correspond to the weight, the deviation from the target, the actual coordinates, and the target coordinates of the *i*-th monitoring point, respectively. The actual coordinates are provided by finite element analysis. Since the focus of this paper is primarily on experimental techniques, the process of finite element analysis is not the emphasis of this study, and thus will not be elaborated upon here.

Regarding the weight issue in the weighted least squares error, this paper adheres to the bilinear law, with the function expression shown in Equation (1). In Equation (2), *s* denotes the normalized curve length along the circumference direction of the profile.

(2) $$\left\{\begin{array}{ll} w=0.3+0.66667s & for\;0<s\leq 0.6\\ w=1.3-s & for\;0.6<s\leq 1 \end{array}\right.$$

The deformation of the morphing wing is directly related to the displacement of the deformation-driven actuation cylinders, and the displacement of these cylinders leads directly to the bending of the variable-camber leading edge. Since the anisotropy of the material has a minimal effect on the deformation, this paper assumes that the deformation of the morphing wing is only related to the displacement of the deformation-driven actuation cylinders, temporarily disregarding other factors. Based on this, it can be considered that the relationship between the maximum strain of the variable-camber leading edge skin and the curvature and thickness at that point is as shown in Equation (3).

(3) $$t\left(s\right)=\frac{\varepsilon_{\lim}}{1/2\Delta \kappa \left(s\right)}$$

In the equation, *ε*_lim_ represents the maximum strain value, *t* is the maximum thickness, and Δ*κ* is the curvature change.

Based on the above analysis, the theoretical model of the target shape for the morphing wing leading edge can be represented by Equation (4).

(4) $$\begin{array}{l} \min\;WLSE=\sum_{i=1}^{n}w_{i}\frac{d_{i}}{n}=\sum_{i=1}^{n}w_{i}\frac{\sqrt{{\left(x_{i}-{x_{i}}^{*}\right)}^{2}+{\left(y_{i}-{y_{i}}^{*}\right)}^{2}}}{n}\\ s.t.:\;K\left(t\right)U\left(t\right)=F\left(t\right)\\ {s_{i}}^{L}<s_{i}<{s_{i}}^{U},i=1,\ldots ,4\\ {f_{xi}}^{L}<f_{xi}<{f_{xi}}^{U},{f_{yi}}^{L}<f_{yi}<{f_{yi}}^{U},i=1,\ldots ,4\\ {t_{ij}}^{L}<t_{ij}<{t_{ij}}^{U},i=1,\ldots ,4,j=1,\ldots ,10 \end{array}$$

In the equation, *K*(*t*) represents the stiffness matrix of the morphing wing; *U*(*t*) and *F*(*t*) are the nodal displacement vector and nodal force vector caused by geometric non-linearity, respectively; *s_i_* is the position of the *i*-th stringer along the skin contour (expressed in terms of a standard length); *s_i_^U^* and *s_i_^L^* are the upper and lower limit positions of the *i*-th stringer, respectively; and *f_xi_* and *f_yi_* are the loads applied in the x and y directions on the *i*-th stringer. (*f_xi_^L^*,*f_xi_^U^*) and (*f_yi_^L^*,*f_yi_^U^*) are the upper and lower bounds of the loads applied in the x and y directions on the *i*-th stringer, respectively. *j* denotes the number of discretizations of the morphing wing.

### 2.2. Theoretical Model Solution Algorithm

When confronted with complex design problems that require the simultaneous optimization of continuous and discrete variables, traditional gradient-based optimization methods often fall short, as they tend to find only local optimal solutions. To overcome this limitation, this study employs the Non-dominated Sorting Genetic Algorithm II (NSGA-II), renowned for its efficiency and stability in global searches.

**Figure 2:**
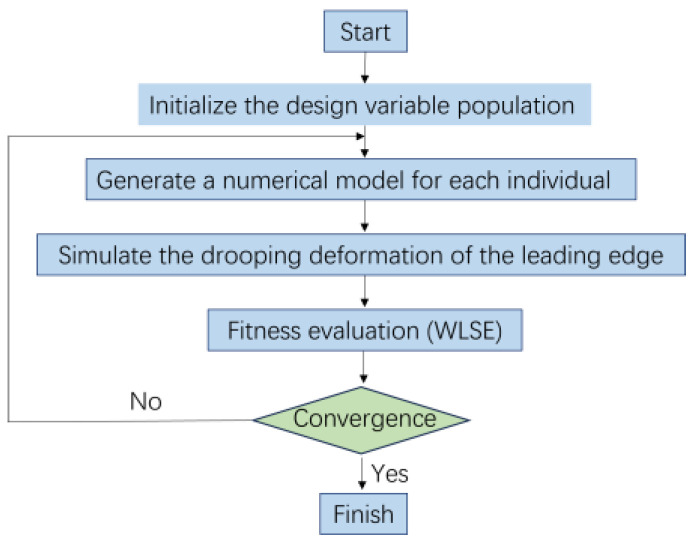
Non-dominated Sorting Genetic Algorithm II.

The process begins by initializing a population of design variables. These design variables encompass the layup sequence of composite materials, the positioning of truss members, and the force components of actuation points, among others. Once the population is initialized, a finite element module generates the corresponding numerical model of the deformed leading edge based on these variables. Subsequently, these numerical models are utilized to simulate the droop deformation of the leading edge. By evaluating the fitness of these models, we can quantify the performance of each design scheme. The fitness assessment employs the weighted least squares error (WLSE) index, aiding in understanding the potential of the model in practical applications. Based on the results of the fitness evaluation, the main program updates the design variables, generating a new generation of design variables through genetic algorithm operations such as selection, crossover, and mutation. This process is repeatedly iterated until a preset convergence criterion is met. The convergence criterion is considered satisfied when the change in the optimal solution and average value of two consecutive generations is less than a threshold of 1.0 × 10^−6^. Following recommendations, the population size is set to four times the number of design variables, with a population size of 200 in this study to ensure adequate search space. The flowchart of the Non-dominated Sorting Genetic Algorithm II (NSGA-II) is shown in Figure 2.

## 3. Test Scheme Design

### 3.1. Study Subjects

**Figure 3:**
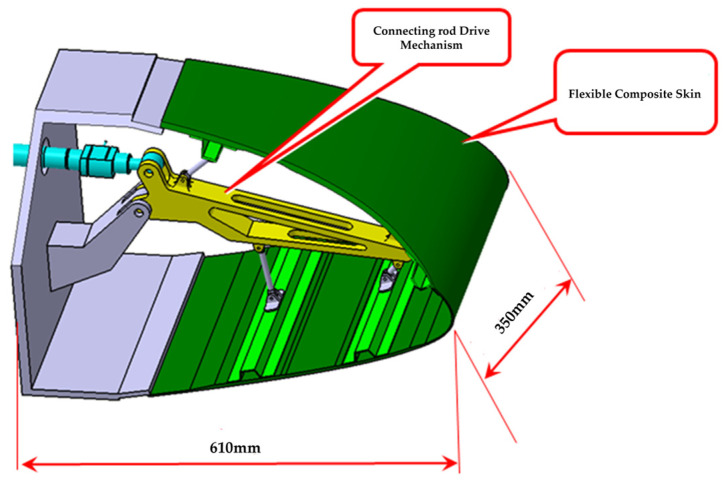
Model of variable-camber wing leading edge structure.

The test piece model of the variable-camber wing leading edge is shown in Figure 3 and consists mainly of a mechanical drive link mechanism and composite fiberglass skin and stringers. There are two main purposes of the test: to verify the motion function of the test piece and monitor the deformation, to verify whether the leading edge test piece meets the design objective of the downward deviation of the aerodynamic shape by 17° and analyze the deformation accuracy; to verify whether the strength of the leading edge test piece meets the design load requirements.

The test differs from the traditional wing test in three main ways. First, when the leading edge is in deflection motion, there is no fixed motion trail, and the motion trail of the test piece is unknown. After converting the aerodynamic load into the test load of the upper and lower wing surface, the motion trail of the upper and lower wing surface load points is also non-linear. Secondly, the test piece is deformed by connecting the mechanical drive mechanism with the deformation drive device, and the upper and lower wing surface load points simulate the flight load. There is a cooperative relationship between the deformation of the test piece and the loading load. Both of these features pose challenges for the precise control of the test and the design of the co-loading. In addition, it is difficult to monitor the deformation of the specimen and reconstruct the shape of the flexible skin because the deformation of the specimen is large during the motion process and the flexible skin of composite materials may be deformed locally or suddenly.

### 3.2. Experimental Device Design

#### 3.2.1. Experimental Loading Device Design

**Figure 4:**
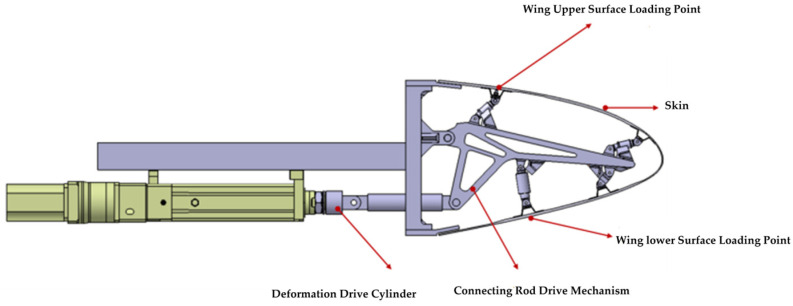
Location of loading points.

**Figure 5:**
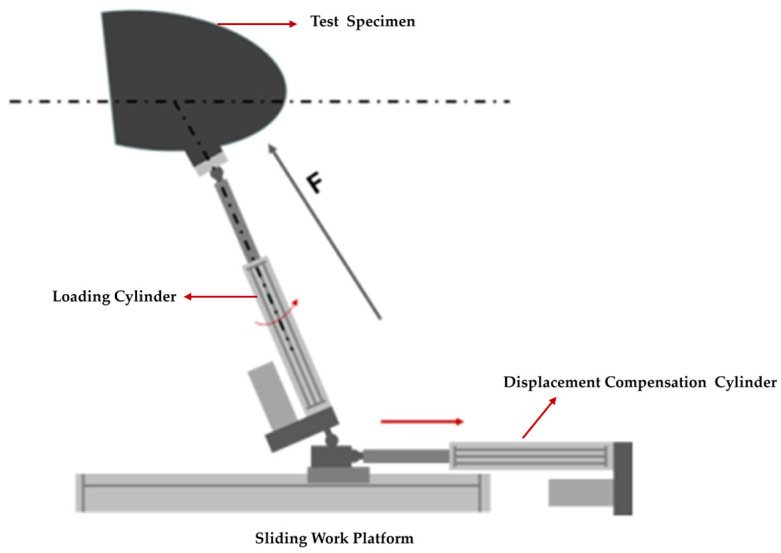
Schematic diagram of follow-up loading device.

**Figure 6:**
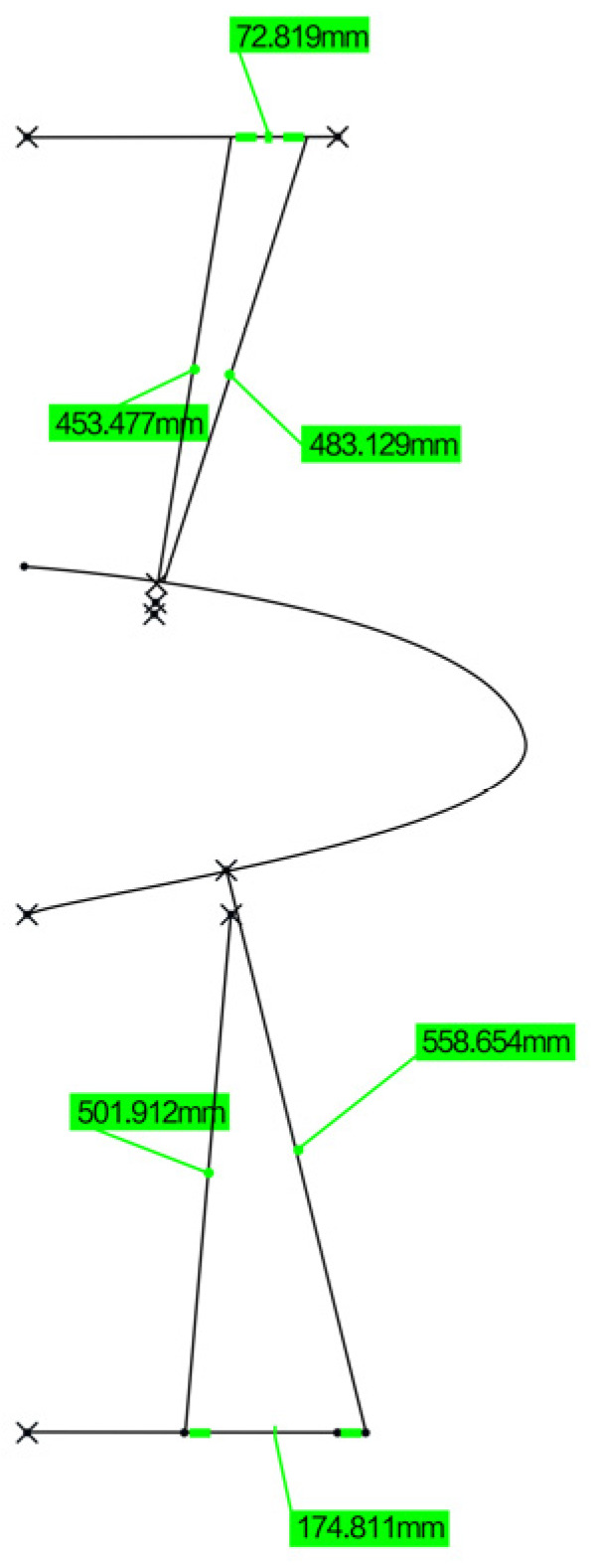
Motion envelope analysis.

In order to solve the loading difficulty caused by the unknown motion trail of the specimen and the non-linearity of the motion trail of the loading point, the loading point of the specimen was determined to be the skin surface corresponding to the upper and lower wing surfaces near the first stringer at the root by finite element analysis, as shown in Figure 4. Based on the principle of the vector loading method, a follow-on loading device was designed, as shown in Figure 5; the loading direction of the loading cylinder was adjusted through the expansion of the displacement compensating cylinder along the normal direction of the skin in real time. The kinematic model was established to analyze the movement envelope range of the specimen, and the movement range of the loading point was calculated, as shown in Figure 6. Based on this, the stroke of the displacement compensating cylinder and the loading cylinder were selected, and the motion trail of the loading point of the upper and lower wing surfaces was fitted.

**Figure 7:**
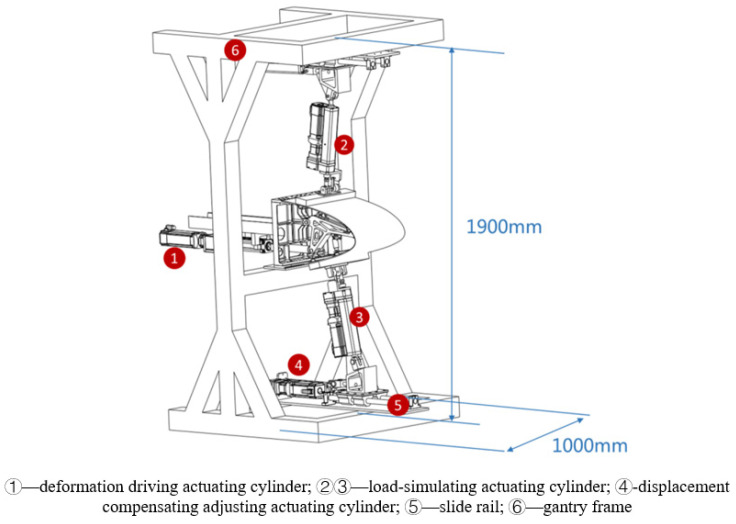
Variable-camber wing leading edge follow-up loading test device.

**Figure 8:**
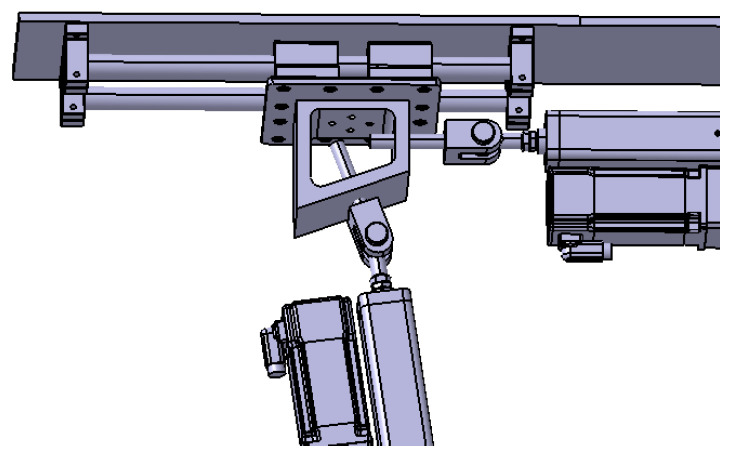
Structure of slide rail.

Based on the above analysis, the design of the variable-camber wing leading edge strength test device is shown in Figure 7. The deformation-driving actuating cylinder is connected to the link mechanism by a hinge and makes a reciprocating motion in the horizontal direction, and drives the link mechanism to drive the skin to realize the leading edge deflection. The loading points of the upper wing surface and the lower wing surface are connected to one end of the loading actuating cylinder. The gantry frame is used to fix the test specimen and the loading equipment, and the gantry frame is fixed on the ground by connecting the ground rail and with the anchor bolts. As shown in Figure 8, a group of slide rail structures is arranged on the upper and lower sides of the gantry frame, respectively; each group of slide rail structures consists of two slide rods and four slide blocks, and ball bearings are built in the slide blocks to ensure smooth movement without sticking; one end of the slide rail is connected to the displacement compensation adjusting actuating cylinder to make it extend and contract in the direction parallel to the slide rail, and the other end is connected to the loading cylinder to adjust the loading direction by way of displacement compensation to the adjusting actuating cylinder.

#### 3.2.2. Loading Control Equipment Design

**Figure 9:**
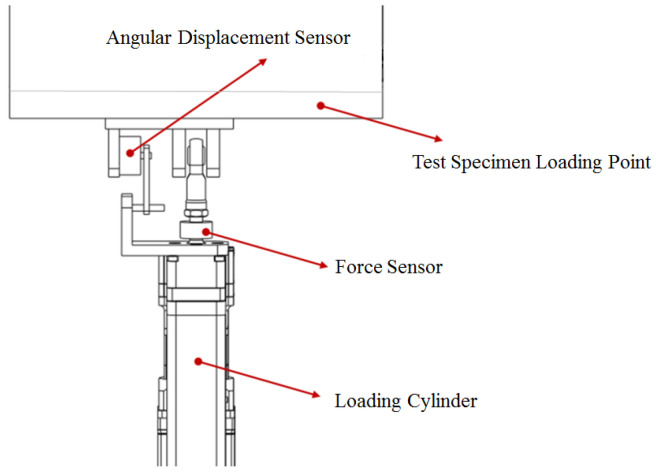
Schematic diagram of sensor setup for test loading points.

**Figure 10:**
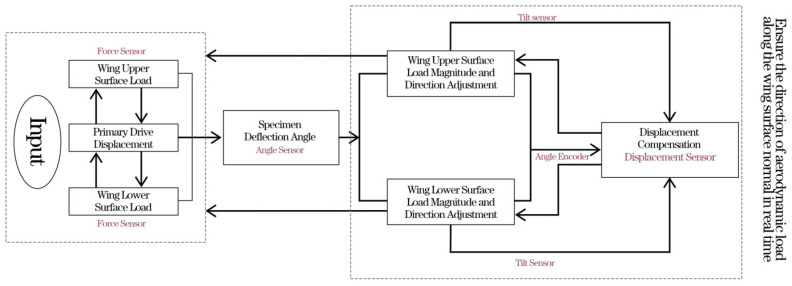
Principle of multi-point cooperative closed-loop control.

In the test, it is necessary to control the deformation-driving actuating cylinder, the load-simulating actuating cylinder, and the displacement-compensating adjusting actuating cylinder to make the deformation of the test piece correspond to the aerodynamic load in real time, and the load direction is along the normal direction of the wing surface in real time. The specific operation begins by applying a suitable small load to the experimental equipment. Based on feedback from various sensors and the experimental setup, the preload is gradually increased until the gaps between all connecting components of the experimental setup are eliminated, ensuring that the load on the test specimen is zero. At this point, the numerical values of the sensors within the control system interface are set to zero to ensure the accuracy of the equipment loading. In order to solve the control problem, a piece of angle-measuring instrument was set on the driving link of the test piece to record the deflection angle of the test piece in real time. The deformation-driving cylinder adopts position control to output the deformation amount in real time, and a functional relationship is established between the displacement amount of the deformation-driving actuating cylinder output and the deflection angle of the test piece. The loading actuating cylinder adopts force control; an angular displacement sensor and a force sensor are arranged between the loading actuating cylinder and the loading point as shown in Figure 9; the angular displacement sensor converts and outputs the angle between the loading cylinder and the wing surface in real time; and the force sensor outputs the actual load in real time. The displacement compensation adjusting actuating cylinder adopts position control, adopts a displacement compensation mode to adjust the loading direction in real time, and performs closed-loop control between the loading angle and the loading direction. A multi-point cooperative closed-loop control system is developed; the motion trail of a test piece obtained by kinematics simulation is used as the initial value input to the control system; and the output of the control system automatically tracks the input amount by iterative mode, reduces the tracking error, improves the control accuracy, suppresses the influence of disturbance signals, ensures the accuracy of the test control loading and realizes the real-time data acquisition and real-time output of the curve. The closed-loop control principle of the test is shown in Figure 10.

### 3.3. Experimental Monitoring Equipment Design

**Figure 11:**
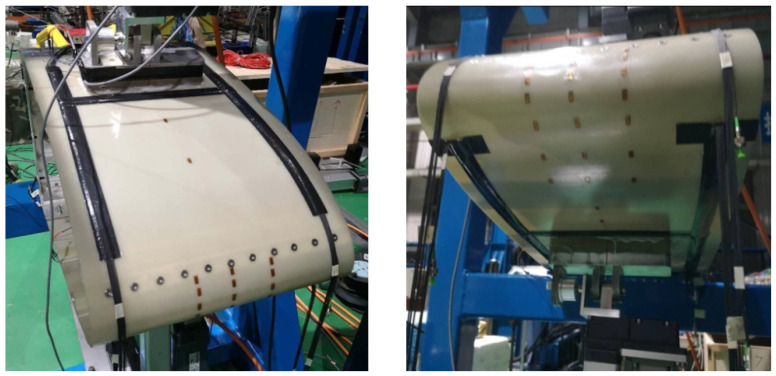
FBG sensor attachment during test.

**Figure 12:**
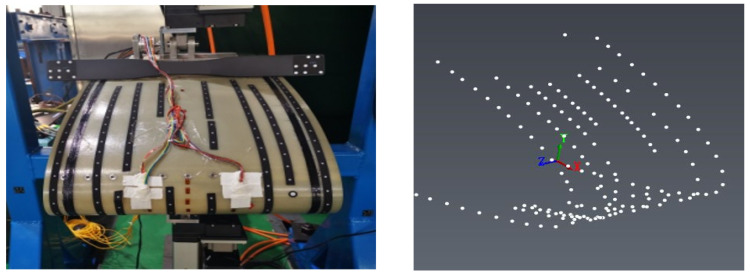
Photogrammetric system working scenario.

**Figure 13:**
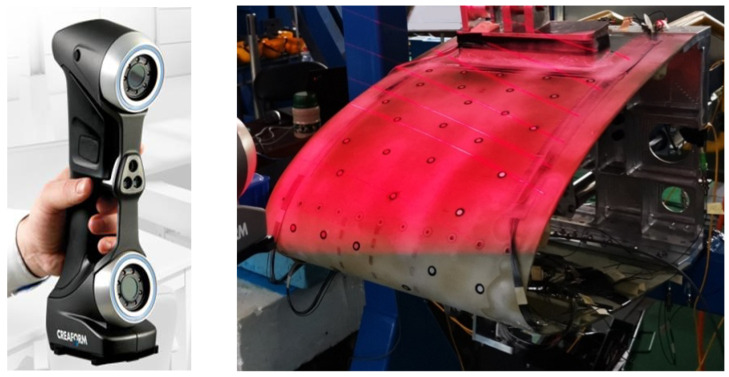
Schematic diagram of 3D rapid-scanning system.

To address the needs of motion deformation measurement and control for the test specimen, considering the characteristics of large deformation of the flexible skin and local abrupt deformation, a complementary sensor monitoring network has been designed. This design is based on the assumption that the deformation of the variable-camber wing along the span direction during the experiment is symmetrical, requiring only the consideration and measurement of deformation within the chord plane. Based on the aforementioned assumption, the shape of the variable-camber wing leading edge is first reconstructed using fiber optic sensors, as shown in the application scenario of Figure 11, where two symmetrical planes are selected in the chord direction for the layout of the fiber optic sensors. Secondly, to verify the validity of the assumption, photogrammetry and three-dimensional rapid scanning techniques were used. In photogrammetry, multiple planes along the span direction are selected to measure the deformation of the variable-camber wing, thereby verifying whether the deformation along the span direction is symmetrical. The application scenario of the photogrammetry system in the experiment is shown in Figure 12. Additionally, the photogrammetry system can also provide supplementary explanations for the measurement results, for comparison with the FBG sensors. The scheme proposed in Figure 13 is a three-dimensional rapid-scanning method, which is a three-dimensional measurement scheme covering the entire span and chord directions of the deforming wing. This is used to verify the assumption and provide supplementary explanations for the measurement results.

This experimental technique offers multiple approaches for measuring the deformation of the morphing wing, extending from two-dimensional to three-dimensional assessments. These approaches are not only comparable with each other to verify their feasibility but also serve to validate the hypotheses proposed in this paper. The use of various measurement methods is intended to ensure the reliability of the results, thereby enhancing the persuasiveness of the experiment.

## 4. Leading Edge Strength Test of Variable-Camber Wing

### 4.1. Test Piece

The leading edge test piece model is derived from a particular type of remote aircraft. The chord of this model is 610 mm derived from the leading edge of the aircraft, and its span is 350 mm. The design target of the aerodynamic deflection angle of the test piece is 17.5°, which corresponds to the maximum downward deflection of the leading edge structure of 15°. The deflection angle in the test is the deflection angle of the leading edge structure.

### 4.2. Test Load

The test load of the leading edge of the variable-camber wing depends mainly on the design and application of the aircraft. The test piece is deformed by driving cylinders in the motion function test and the upper and lower wing surfaces are not loaded. The strength test load is derived from the results of the aerodynamic analysis, which is equivalent to the test load applied to the upper and lower wing surfaces. The corresponding relationship between deformation-driving cylinder displacement, structural deformation angle, and test load is shown in Table 1.

Regarding the data in Table 1, since the deformation of the specimen in the motion function test is caused by the actuation of the driving cylinder, the generation of the deflection angle is due to the bending of the variable-camber wing leading edge. Therefore, the deflection angle of the wing is directly proportional to the displacement. Observations from Table 1 of deflection angles and displacements reveal that the increase in displacement exhibits a linear relationship with the increase in deflection angle. In the current working condition, the strain is minimal and the deforming wing is in the elastic phase, which means that stress and strain are in a linear relationship. This leads to a linear relationship between the deflection angle and stress as well. Consequently, when plotting the data from Table 1 in subsequent graphs, lines should be used to connect the points.

### 4.3. Test Results of Loading Condition

**Figure 14:**
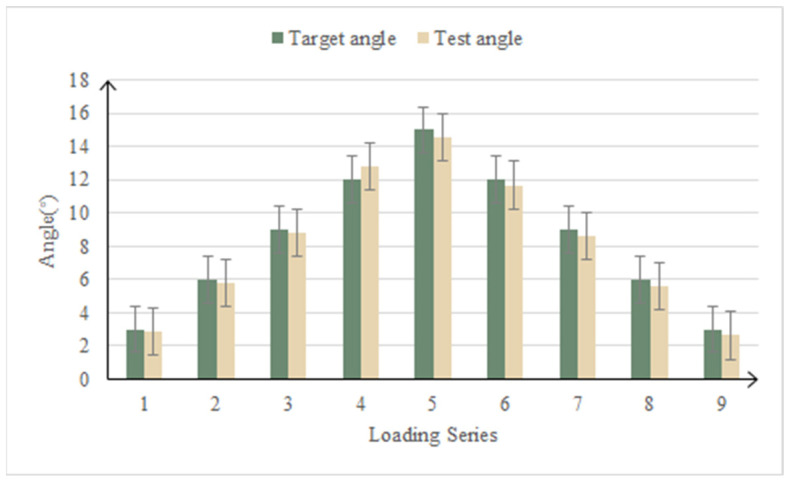
Contrast curve of deflection angle in leading edge motion function test.

In the motion function test, the loading point of the upper and lower wing surface was followed-up, the load was always kept at zero, the deformation-driving cylinder drove the test piece to deflect, and the error between the actual deformation of the test piece and the design theoretical value was the main focus. The contrast curve of the measured deflection angle and the theoretical deflection angle is shown in Figure 14. The average control error of the deflection angle in the function test of the leading edge motion of the variable-camber wing is 4.59%.

**Figure 15:**
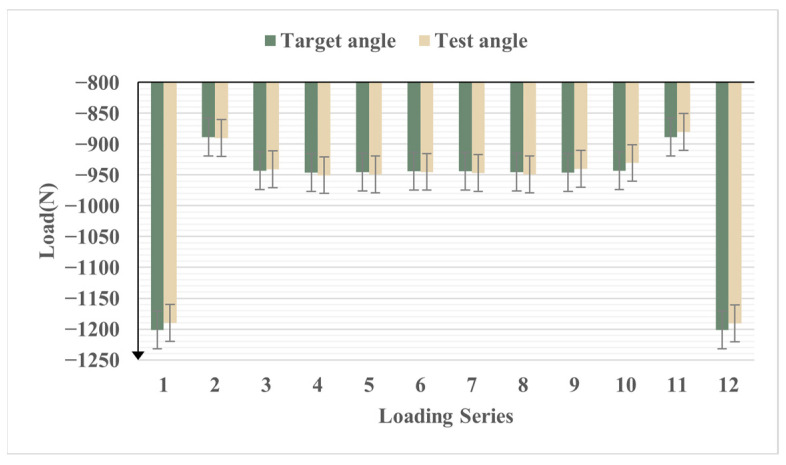
Contrast curve of load on wing surface at leading edge strength test.

**Figure 16:**
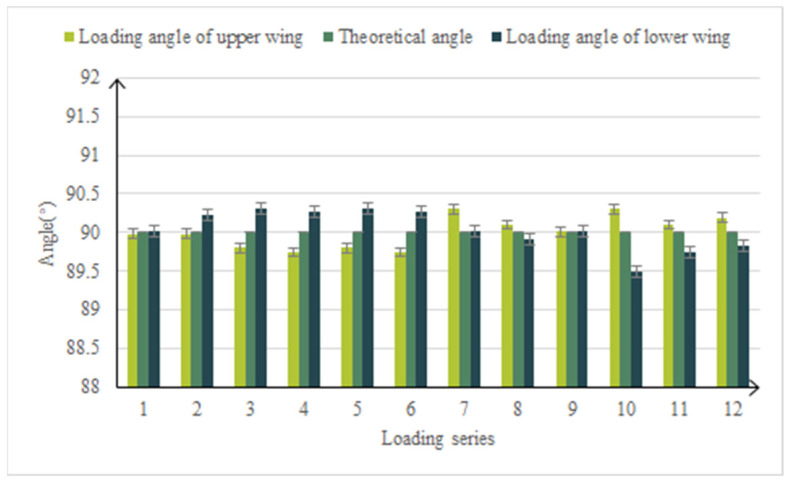
Contrast curve of load angle between upper and lower wing surface during leading edge strength test.

In the strength verification test, the upper and lower wing load points followed the dynamic load, and the load direction was in real time along the normal direction of the wing surface. The load of the upper wing surface is large and is the main loading point. The average error of the upper surface load is 0.54%. The contrast curve of the load is shown in Figure 15. The average error of the load applied angle is 0.24% and the comparison curve of the load angle is shown in Figure 16. It can be seen that the variable-camber wing leading edge test device accurately simulated the deformation process of the leading edge under real flight and driving loads.

### 4.4. Test Results and Analysis

**Figure 17:**
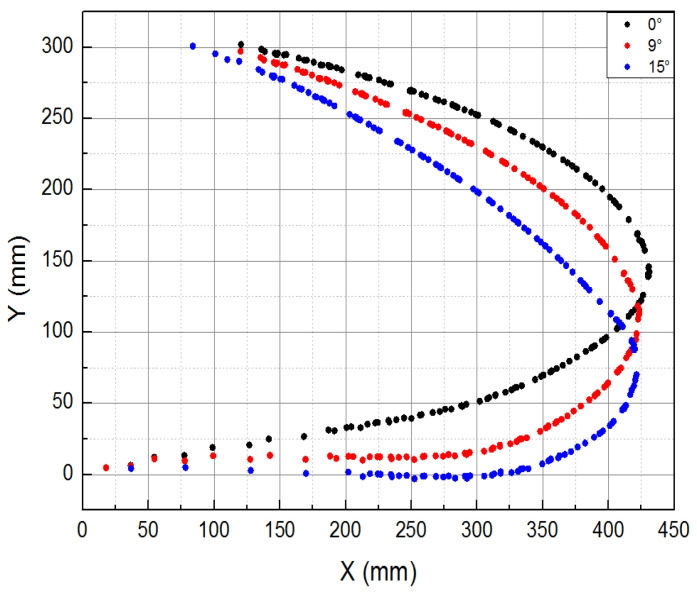
Shape change curve of leading edge in motion function test (0°, 9°, 15°).

**Figure 18:**
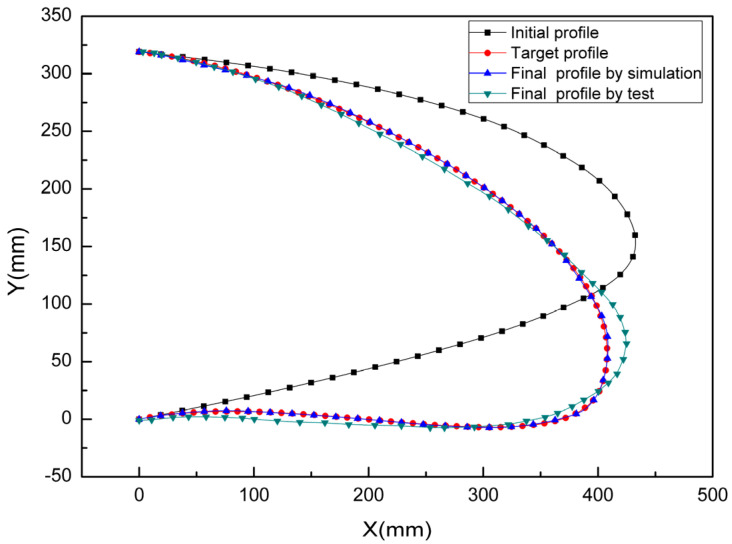
Analysis of deformation accuracy of leading edge in motion function test.

During the motion function test, the deformation of the composite material skin was smooth and continuous, with no delamination observed at the connection points between the skin and the truss members. All connection parts of the test specimen functioned normally, without any abnormal noise. The mechanical linkage transmission structure deformed smoothly, with no jamming phenomena. Figure 17 and Figure 18, respectively, show the shape change curve of the leading edge and the deformation accuracy curve when the leading edge structure is lowered by 15% during the motion function test. As the test specimen deforms through the actuation of the driving cylinder, displacement is generated to achieve the deflection of the flap. Consequently, as the downward deflection angle of the leading edge structure increases, the deformation at the tip of the leading edge also gradually increases. The greater the deflection angle, the lower the leading edge structure of the deformed wing. It can be observed that a downward adjustment of 17° for the leading edge meets the aerodynamic shape design target, and the maximum difference between the actual deformation and theoretical deformation at the tip of the leading edge occurs when the leading edge structure is lowered by 15° (aerodynamic shape adjustment of 17.5°), with the maximum deformation error being less than 10 mm.

**Figure 19:**
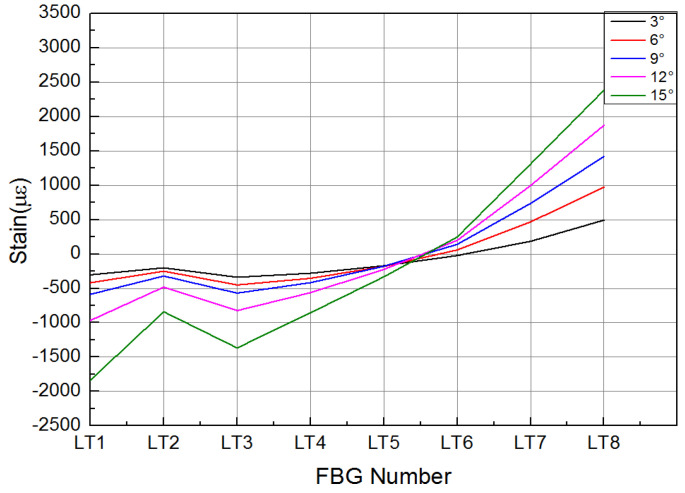
Strain curve of wing upper surface with different deflection angle of leading edge.

**Figure 20:**
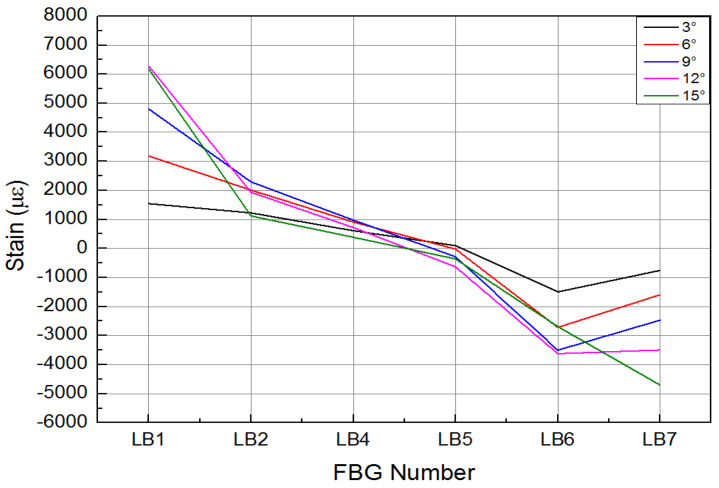
Strain curve of wing lower surface with different deflection angle of leading edge.

**Figure 21:**
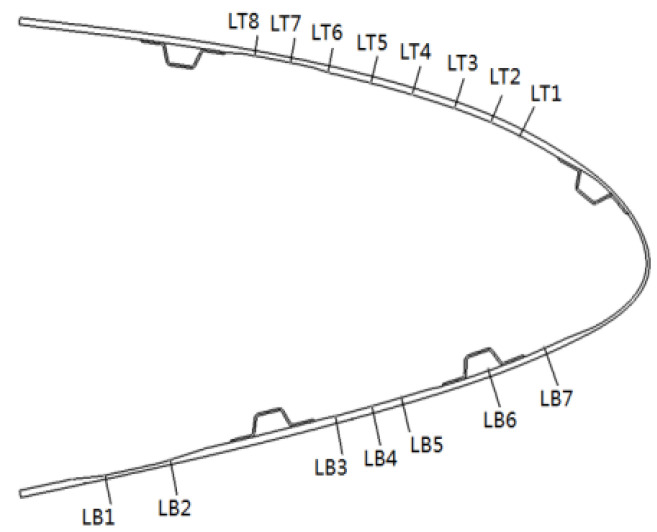
FBG sensor arrangement.

In the strength validation test, the deformation of the composite material skin was smooth and continuous, with no delamination at the connection points between the skin and the truss members, and no abnormal noise from any connection parts of the specimen. The mechanical linkage transmission structure deformed smoothly, with no jamming phenomena, and no structural damage occurred. Figure 19 and Figure 20, respectively, show the strain curves of the upper and lower wing surfaces under different leading edge deflection angles. Figure 21 illustrates the placement positions of the FBG (Fiber Bragg Grating) fiber optic sensors. Specifically, LB1 to LB7 denote the locations of the FBG sensors on the upper wing surface, while LT1 to LT8 indicate the positions where the FBG sensors are arranged on the lower wing surface. According to the FBG sensor layout plan in Figure 21, the upper wing surface near the wing tip is in a state of compression, while the upper wing surface near the wing root is in a state of tension, and both increase with the increase in deflection angle. The lower wing surface near the wing root is in a state of tension, and the lower wing surface near the wing tip is in a state of compression, with strains increasing as the deflection angle increases, and the strain on the lower wing surface being greater than that on the upper wing surface. The maximum tensile and compressive strains both occur at the maximum deflection angle (15°), with the maximum tensile strain being 6284 με and the maximum compressive strain being 4698 με. Additionally, it can be seen from the figures that the greater the deflection angle, the greater the slope of the strain curve. This is because the strain at the FBG sensor location on the deforming wing is caused by the stress the deforming wing undergoes; thus, the slope of the strain curve at the FBG sensor represents the stress at that location. Therefore, the greater the deflection angle of the deforming wing, the greater the deformation of the leading edge components, resulting in greater stress and, consequently, a steeper slope of the strain curve.

**Figure 22:**
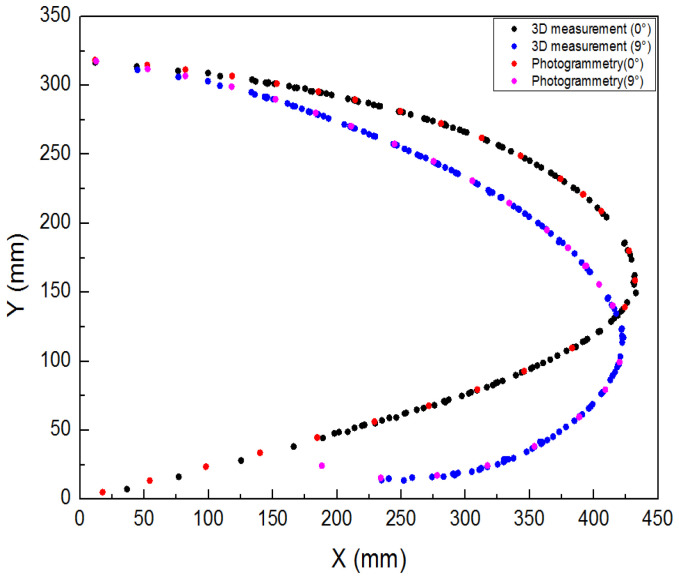
Contrast curve of 3D scanning and photogrammetry results (0°, 9°).

**Figure 23:**
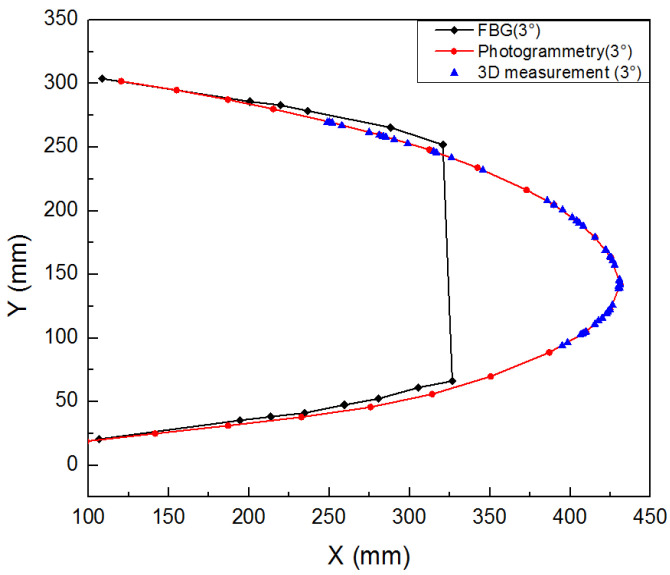
FBG/3D scanning/photogrammetry results (3°).

Three different measurement methods were compared and analyzed. As shown in Figure 22, the average error between the 3D scan and photogrammetric results was less than 1% when the leading edge structure was deflected downward by 0° and 9°. As shown in Figure 23, when the leading edge structure was deflected downward by 3°, the average relative error of the FBG measurement, photogrammetry, and 3D fast scan method was 8.88%. During the test, when the downward deflection angle of the leading edge structure was more than 3°, the FBG sensors at the wing tip position were all ineffective. The results show that the photogrammetry and 3D scanning methods have high measurement accuracy, but are affected by the test conditions, such as light and occlusion. Thus, the real-time measurement methods based on the above two methods can be further studied and applied to the test of the morphing wing. The FBG method can monitor the movement process of the variable-camber wing in real time, but it is affected by the sensor type, adhesive technology, and data processing method for shape reconstruction. The problems such as the lack of test data and the measurement error caused by the above factors need to be solved later.

## 5. Conclusions

1. The variable-camber wing leading edge is an aeronautical design that mimics the continuous and smooth form of a bird’s wings. In response to the variable-camber wing, this paper introduces a ground-based strength testing technique for the variable-camber wing leading edge. This technique effectively fills the technical gap in monitoring full-scale variable-camber wing leading edges under flight conditions, providing technical support for the testing and evaluation of the leading edge.
2. The deformation process of the leading edge of a full-scale variable-camber wing under real flight load and drive load was accurately simulated. The test results show that the motion function and bearing capacity of the leading edge structure meet the design requirements, and the average deflection angle error is 4.59%.
3. A multi-point cooperative control system with precise control, fast response, and stable operation was developed. The feedback results show that the control frequency of the system is as high as 1000 Hz, and the average error of the applied load magnitude and direction is 0.54% and 0.24%, respectively.
4. The distributed sensor monitoring network was reasonably designed to ensure that the entire motion process of the leading edge can be measured and controlled. The measurement results show that the maximum error between the actual deformation and the theoretical deformation is less than 10 mm, and the design target deflection angle was realized.
5. While the present study has provided valuable technical support for the testing and evaluation of variable-camber wing leading edges, there are limitations due to manufacturing processes that have restricted the experimental techniques to full-scale measurements in the chordwise direction only, without encompassing the full spanwise extent. Consequently, to more effectively assess the condition of the variable-camber wing system, future endeavors could explore the implementation of distributed control systems to conduct ground strength tests that encompass both the full spanwise and chordwise dimensions.

## Figures and Tables

**Table 1:** Test load of variable-camber wing leading edge.

Structure Deflection Angle (°)	Deformation-Driving Actuating Cylinder Displacement (mm)	Wing Upper Surface Test Load (*n*)	Wing Lower Surface Test Load (*n*)
0	0	−1201.26	61.28
3	−7.30	−888.99	90.39
6	−14.63	−943.49	92.079
9	−21.96	−946.21	104.27
12	−29.26	−945.18	106.95
15	−36.35	−944.15	109.63

## Data Availability

Data are contained within the article.
